# Transporter-Driven Engineering of a Genetic Biosensor for the Detection and Production of Short-Branched Chain Fatty Acids in *Saccharomyces cerevisiae*

**DOI:** 10.3389/fbioe.2022.838732

**Published:** 2022-03-18

**Authors:** Ryoma Miyake, Hua Ling, Jee Loon Foo, Nobutake Fugono, Matthew Wook Chang

**Affiliations:** ^1^ NUS Synthetic Biology for Clinical and Technological Innovation (SynCTI), National University of Singapore, Singapore, Singapore; ^2^ Synthetic Biology Translational Research Programme, Yong Loo Lin School of Medicine, National University of Singapore, Singapore, Singapore; ^3^ Department of Biochemistry, Yong Loo Lin School of Medicine, National University of Singapore, Singapore, Singapore; ^4^ Science & Innovation Center, Mitsubishi Chemical Corporation, Yokohama, Japan

**Keywords:** short branched-chain fatty acids, weak organic acids, transporter, genetic biosensor, promoter, *Saccharomyces cerevisiae*

## Abstract

Biosensors can be used for real-time monitoring of metabolites and high-throughput screening of producer strains. Use of biosensors has facilitated strain engineering to efficiently produce value-added compounds. Following our recent work on the production of short branched-chain fatty acids (SBCFAs) in engineered *Saccharomyces cerevisiae*, here we harnessed a weak organic acid transporter Pdr12p, engineered a whole-cell biosensor to detect exogenous and intracellular SBCFAs and optimized the biosensor’s performance by varying *PDR12* expression. We firstly constructed the biosensor and evaluated its response to a range of short-chain carboxylic acids. Next, we optimized its sensitivity and operational range by deletion and overexpression of *PDR12*. We found that the biosensor responded to exogenous SBCFAs including isovaleric acid, isobutyric acid and 2-methylbutanoic acid. *PDR12* deletion enhanced the biosensor’s sensitivity to isovaleric acid at a low concentration and *PDR12* overexpression shifted the operational range towards a higher concentration. Lastly, the deletion of *PDR12* improved the biosensor’s sensitivity to the SBCFAs produced in our previously engineered SBCFA-overproducing strain. To our knowledge, our work represents the first study on employing an ATP-binding-cassette transporter to engineer a transcription-factor-based genetic biosensor for sensing SBCFAs in *S. cerevisiae*. Our findings provide useful insights into SBCFA detection by a genetic biosensor that will facilitate the screening of SBCFA-overproducing strains.

## Introduction

Carboxylic acids are used as platform chemicals for manufacturing a variety of industrial products such as plastics, solvents, polymers and serve as “building blocks” for pharmaceuticals, fragrances, foods and other valuable products (Sauer et al., 2008). In the industry, carboxylic acids are produced from petrochemicals, which has brought concerns about unsustainability and environmental pollution. Therefore, there are strong interests in developing economical and environmentally friendly routes to produce carboxylic acids using microbial hosts (Chen and Nielsen, 2016). Isovaleric acid (IVA), isobutyric acid (IBA) and 2-methylbutanoic acid (2MBA) are representative short branched-chain fatty acids (SBCFAs) containing a methyl branch located on one or two carbon atoms (Yu et al., 2016). IVA derivatives are used as drugs, e.g., anticonvulsant and sedative and flavourings (Suerbaev et al., 2012; Wang et al., 2018). IBA derivatives are used as plastics, flavorings, fragrances, textile auxiliaries, surfactants and plasticizers (Zhang et al., 2011; Lang et al., 2014; Hammer et al., 2020). 2MBA is used for the synthesis of pharmaceuticals and flavoring (Kwon et al., 2000). Recently, several microorganisms, including *Saccharomyces cerevisiae*, *Pseudomonas putida* and *Bacillus licheniformis*, have been engineered to produce these SBCFAs (Lang et al., 2014; Yu et al., 2016; Shi et al., 2019). However, their production level in the engineered microbes is still low.

Genetic biosensors can be developed and used to facilitate strain engineering and optimization by biosensor-assisted high-throughput screening (HTS) of microbes for high SBCFA producers. Typically, a genetic biosensor consists of an allosterically regulated transcription factor (TF), which binds a specific ligand and associates with a target promoter to regulate the expression of a reporter gene (Teo and Chang, 2015; Taylor et al., 2016; Chen et al., 2018; Xia et al., 2019; D'Ambrosio et al., 2020; Hossain et al., 2020). For constructing an SBCFA biosensor, the weak organic acid-activated TF, War1p, from *S. cerevisiae* is a suitable candidate. War1p is constitutively expressed and binds to the weak organic acid response (War) element in the *PDR12* promoter (pPDR12) (Kren et al., 2003; Gregori et al., 2008). When it binds to a weak organic acid, War1p is phosphorylated and conformationally changed to activate the expression of *PDR12* (Mollapour and Piper, 2012). Based on this inducible system, Baumann et al. have developed a genetic biosensor which consists of War1p and pPDR12 coupled with green fluorescent protein (GFP) to detect short- and medium-chain fatty acids in *S. cerevisiae*, in which the fatty acid sensing was decoupled from the production using a two-cell sensor system (Baumann et al., 2018). Compared to the two-cell sensor system, a one-cell sensor system that produces and senses SBCFAs is favored for HTS of SBCFA-producing strains (Lim et al., 2018).

Optimizations are commonly needed to achieve desired properties of the genetic biosensors such as dynamic and operational ranges for the effective detection of target compounds (Hossain et al., 2020). The main strategies for such optimization include tuning of the TF’s receptor level, engineering of the DNA binding domain, the promoter’s upstream region, operator sequence or the 5′-untranslated region of the reporter gene and control of the target chemical level. For instance, Snoek et al. have reported that the evolution-guided engineering of the ligand binding domain of a bacterial TF (BenM) in *S. cerevisiae* led to a 15-fold increase in sensitivity and a 40-fold change in operational range (Snoek et al., 2020). Dabirian et al. have inserted the binding sites into various positions in the core promoter region of five native promoters in *S. cerevisiae*, which led to a higher sensitivity of malonyl-CoA-responsive TF (FapR) (Dabirian et al., 2019). Williams et al. have developed a pPDR12-based genetic biosensor to detect p-hydroxybenzoic acid and propionic acid through a positive-feedback activation of War1p by promoter engineering (Williams et al., 2017). Reduction of the intracellular level of target chemical by activating an exporter shifts the operational range of an allosteric TF-based biosensor in *E. coli* (Raman et al., 2014). In yeast, the ATP-binding-cassette (ABC) transporter Pdr12p plays roles in regulating the intracellular level of weak organic acids by export and its expression is selectively induced by weak organic acids (Piper et al., 1998; Hatzixanthis et al., 2003). Therefore, we hypothesized that tuning of *PDR12* expression could be employed to optimize the properties of genetic biosensors for the detection of weak organic acids such as SBCFAs.

In this study, we aimed to develop a genetic biosensor to detect SBCFAs (exemplified by IVA, IBA and 2MBA) produced by our previously engineered *S. cerevisiae* strain (Yu et al., 2016). To this end, we firstly constructed a biosensor based on the War1p TF and pPDR12 and characterized its substrate spectrum. Furthermore, we optimized its sensitivity and operational range by deletion and overexpression of *PDR12* in the engineered SBCFA-overproducing strain. This study provides useful insights into SBCFA detection by a one-cell biosensor system that can potentially facilitate the HTS of SBCFA-overproducing strains.

## Materials and Methods

### Plasmids and Strains

Plasmids and strains used in this study are shown in Table 1. Oligonucleotide primers (Supplementary Table S1) were synthesized by Integrated DNA Technologies (Singapore). Chemically competent *Escherichia coli* TOP10 was used for subcloning. The transformants were selected on LB agar plates with 100 μg/mL ampicillin.

**TABLE 1:** Plasmids and strains used in this study.

Plasmids/Strains	Description	Source
Plasmids		
pESC-URA	AmpR, URA3, 2-micron ori	Agilent
pUG72	AmpR, *loxP–URA3–loxP*	Gueldener et al. (2002)
pUG72-TEF1	pUG72 carrying P_TEF1_	Yu et al. (2016)
pSH69	AmpR, hpnMX, Cre under P_GAL1_ control	Hegemann and Heick, (2011)
pPDR12-GFP	pESC-URA carrying P_PDR12_ and yEGFP	This study
Strains		
*E. coli*		
*E. coli* TOP10	F− *mcrA* Δ (*mrr-hsdRMS-mcrBC*) ϕ80*lacZ*ΔM15 ΔlacX74 recA1 *araD*139 Δ(*ara-leu*)7697 *galU galK rpsL*(*Str* ^*R*^) *endA1 nupG*	Invitrogen
*S. cerevisiae*		
BY4741	MATa *his3*Δ*1 leu2*Δ*0 met15*Δ*0 ura3*Δ*0*	ATCC
4G-ΔADH6	BY4741 with overexpressed *BAT1*, *ARO10*, *ALD2*, *ALD5* and disrupted *ADH6*	Yu et al. (2016)
TOE01	BY4741 with overexpressed *PDR12*	This study
TDL01	BY4741 with disrupted *PDR12*	This study
TDL02	4G-ΔADH6 with disrupted *PDR12*	This study
BY4741-S_LCA_	BY4741 with pPDR12-GFP	This study
4G-ΔADH6-S_LCA_	4G-ΔADH6 with pPDR12-GFP	This study
TOE01-S_LCA_	TOE01 with pPDR12-GFP	This study
TDL01-S_LCA_	TDL01 with pPDR12-GFP	This study
TDL02-S_LCA_	TDL02 with pPDR12-GFP	This study

### Chemicals and Reagents

The Q5 high-fidelity DNA polymerase, NEBuilder HiFi DNA Assembly Cloning Kit, Restriction enzymes, T4 DNA ligase and PCR reagents were purchased from New England Biolabs (Beverly, MA, United States). QIAquick Gel Extraction Kit, QIAprep Spin Miniprep Kit and RNeasy Mini Kit were purchased from Qiagen (Valencia, CA, United States). Peptone was purchased from Oxoid Ltd. (Basingstoke, Hampshire, UK). Dropout SC amino acid mixture formulation (without Uracil) for *S. cerevisiae* growth media was purchased from MP Biomedicals (Santa Ana, CA, United States). All other reagents were purchased from Sigma Aldrich (St. Louis, MO, United States) or Tokyo Chemical Industry Co., Ltd. (Tokyo, Japan) unless otherwise stated.

### Construction of the SBCFA Biosensor

To construct the SBCFA biosensor, a GFP reporter gene was cloned under the control of pPDR12 which is inducible by weak organic acids. Specifically, pPDR12 was amplified by PCR from the genomic DNA of *S. cerevisiae* BY4741 using primers PDR12p-f with a *Sac*I site and PDR12p-r (Supplementary Table S1). The GFP gene was amplified from pKT127 harboring the yEGFP gene (Accession No. MK178572) (Sheff and Thorn, 2004) using primers yEGFP-f and yEGFP-r with an *Xho*I site. pESC-URA was digested with *Sac*I and *Xho*I. The amplified pPDR12, *yEGFP* and the digested pESC-URA fragment were purified and assembled by NEBuilder to yield the SBCFA biosensor namely pPDR12-GFP. The sequence of the biosensor plasmid was confirmed by Sanger sequencing (1st Base, Singapore).

### Deletion and Overexpression of *PDR12* in *S. cerevisiae*

To tune the expression of *PDR12* gene and study its effect on the biosensor’s behavior, *PDR12* was deleted and over-expressed in *S. cerevisiae*. Briefly, the *PDR12* gene deletion was carried out using the Cre-LoxP system (Gueldener et al., 2002). The plasmid pUG72 (carrying the loxP–*URA3*–loxP cassette) was used to generate the gene deletion cassette and its derivative plasmid namely pUG72-TEF1 (carrying the strong constitutive promoter pTEF1) was used to replace the pPDR12 with pTEF1 in the chromosome (Yu et al., 2016). The plasmid pSH69 (carrying pGAL1-CRE) which contains a hygromycin B resistance gene was used for marker rescue (Hegemann and Heick, 2011).

To delete the *PDR12* gene, the *URA3* selection marker flanked by loxP sites was amplified from pUG72 by PCR using primers PDR12-Del-f and PDR12-Del-r, which contained a 42 bp homology to the integration site of *PDR12* gene (Supplementary Table S1). The obtained DNA fragments were purified and transformed into BY4741 and the SBCFA-overproducing strain 4G-ΔADH6 using a modified yeast transformation method (Gietz and Schiestl, 2007; Ling et al., 2015). The transformants were selected on solid defined medium lacking uracil (SCD-U) and confirmed by colony PCR using the primers PDR12-CheGD-f PDR12-CheGD-r (Supplementary Figure S1). The obtained strains were transformed with the CRE recombinase-expressing plasmid pSH69 and grown on YPD plates containing 200 μg/mL hygromycin B (YPDH) to remove the *URA3* marker gene between the loxP sites. The obtained strains were evaluated by PCR to confirm removal of the *URA3* gene and designated as TDL01 (derived from BY4741) and TDL02 (derived from 4G-ΔADH6).

The replacement of the native promoter of *PDR12* gene (pPDR12) to *TEF1* promoter (pTEF1) was carried out using the same procedure as described for *PDR12* deletion. The pTEF1 was amplified by using primers PDR12-pTEF1-F and PDR12-pTEF1-R and pGU72-TEF1 as a template. The promoter replacement in the obtained transformants were confirmed by PCR with the primers TEFp-UP-f and PDR12-CheTEFp-r (Supplementary Figure S1). After marker rescue, the obtained strains were evaluated by PCR with primers PDR12-CheTEFp-f and PDR12-CheTEFp-r to confirm removal of the *URA3* gene. The confirmed strain was used for transformation with the constructed SBCFA biosensor as described above.

### Detection of Exogenous SBCFAs by the SBCFA Biosensor

Prior to the detection of exogenous SBCFAs, the strains harboring the SBCFA biosensor pPDR12-GFP were grown in SCD-U with 100 mM potassium phosphate buffer (KPB, pH6.5) overnight at 30°C with shaking at 200 rpm. The overnight culture was diluted to OD_600_ of 0.1 in 2 mL of fresh medium and grown until the OD_600_ reached 0.4–0.6. The cells were centrifuged and resuspended in fresh SCD-U with 100 mM KPB (pH6.5) and 100 μL of cells was transferred to each well in black 96-well plates with clear flat bottom. Subsequently, 90 μL of fresh SCD-U with KPB was mixed with 10 μL of SBCFAs (Supplementary Table S2) to final concentrations of 0.05–10 mM, where the SBCFA stocks were prepared at 1 M in 70% ethanol and pH neutralized with sodium hydroxide solution. The SBCFA stocks were diluted to 1–200 mM using sterile water before adding into the microplate wells.

To measure the fluorescence signal of the SBCFA biosensor, the samples described as above in the 96-well plate were incubated at 30°C with shaking at 600 rpm in a Synergy H1 plate reader (BioTek, VT, United States). The cell density (OD_600_) was measured at 600 nm and the fluorescence signal was measured with excitation at 470 nm and emission at 515 nm every 30 min. The fluorescence intensity was normalized to OD_600_ and the normalized fluorescence intensity was compared between the conditions with and without SBCFAs. The EC_50_ (50% effective concentration) values were calculated by nonlinear regression using GraphPad Prism version 9 software (Graph Pad Software, CA, United States).

### Detection of Intracellularly Produced SBCFAs by the SBCFA Biosensor

Besides the detection of exogenous SBCFAs, the intracellular SBCFAs produced by our previous SBCFAs-overproducing strain 4G-ΔADH6 was detected. The strains were cultivated in SCD-U medium at 30°C with shaking at 220 rpm overnight. The overnight culture was re-inoculated to an initial OD_600_ of 0.05 in 25 mL fresh SCD-U with 100 mM KPB (pH6.5) and grown under the same conditions in 250 mL shake flasks. For time course experiments, culture samples were collected every 12 h to determine the SBCFA levels and cell density (OD_600_) was measured by a spectrophotometer BioPhotometer Plus (Eppendorf, Germany). The culture samples were diluted by SCD-U with KPB to adjust the cell concentration and the fluorescence intensity was measured on a Synergy H1 microplate reader and normalized to the cell density (Jung et al., 2021) as described above.

### Gas Chromatography/Mass Spectrometry Analysis of SBCFAs

GC analysis of SBCFAs was performed using methods modified from previous studies (Teo et al., 2015; Yu et al., 2016; Ng et al., 2020). One milliliter of yeast culture was collected and centrifuged to obtain the supernatant. To quantify the SBCFAs in the supernatant, 0.4 mL of 10% hydrochloric acid–methanol (v/v) and heptadecanoic acid (C17:0) at a final concentration of 1 mM as an internal standard were added to 0.8 mL culture supernatant and the mixtures were vortexed for 2 min and incubated at 62°C for 3 h to methylate the SBCFAs. SBCFA standards (IVA, 2MBA, IBA) at 0.5 and 2.0 mM added to SCD-URA medium were extracted and methylated as describe above. To the reaction mixtures, 0.2 mL hexane was added, and the resulting fatty acid methyl esters (FAMEs) were extracted by vortexing for 2 min and phase separation by centrifugation. The organic extracts containing FAMEs were then subjected to GC/MS analysis using a 7890B GC system with an Agilent 5977A MSD and an HP-5MS column. The method for GC/MS analysis was as follows: oven temperature with initial hold at 45°C for 3 min, ramp to 50°C at 10°C/min and hold for 3 min, ramp to 280°C at 50°C/min and hold for 5 min. Helium was used as the carrier gas and set at a constant pressure of 13.8 psi. The injector was maintained at 250°C and the ion source temperature was set to 230°C. The injection volume was 1.0 μL in a splitless mode. FAMEs of the respective SBCFAs were identified by their retention time and the mass spectra of FAME standards. Data analysis was performed using the Agilent Enhanced Data Analysis software. Given that 2MBA and IVA are isomers that have the same mass spectrum and retention time, they were quantified as a mixture.

## Results and Discussion

### Construction and Characterization of the SBCFA Biosensor

We have previously engineered *S. cerevisiae* to produce SBCFAs that comprise IVA, IBA and 2MBA (Yu et al., 2016). Here, to construct a genetic biosensor to detect SBCFAs in *S. cerevisiae*, we designed a biosensor that consists of 1) War1p, a TF that phosphorylates upon binding to weak organic acids (e.g., hexanoic acid, heptanoic acid and octanoic acid), 2) pPDR12, which is activated by the phosphorylated War1p and 3) a yeast-enhanced GFP gene (*yEGFP*) under the control of pPDR12. To ensure a higher copy number of the target genes, we cloned the aforementioned genetic elements into a high-copy plasmid pESC-URA and introduced the resulting biosensor (namely S_LCA_) into *S. cerevisiae* strains (BY4741, TOE01, TDL01, TDL02, 4G-ΔADH6) to obtain the biosensor strains (BY4741-S_LCA_, TOE01-S_LCA_, TDL01-S_LCA_, TDL02-S_LCA_, 4G-ΔADH6-S_LCA_).

**FIGURE 1:**
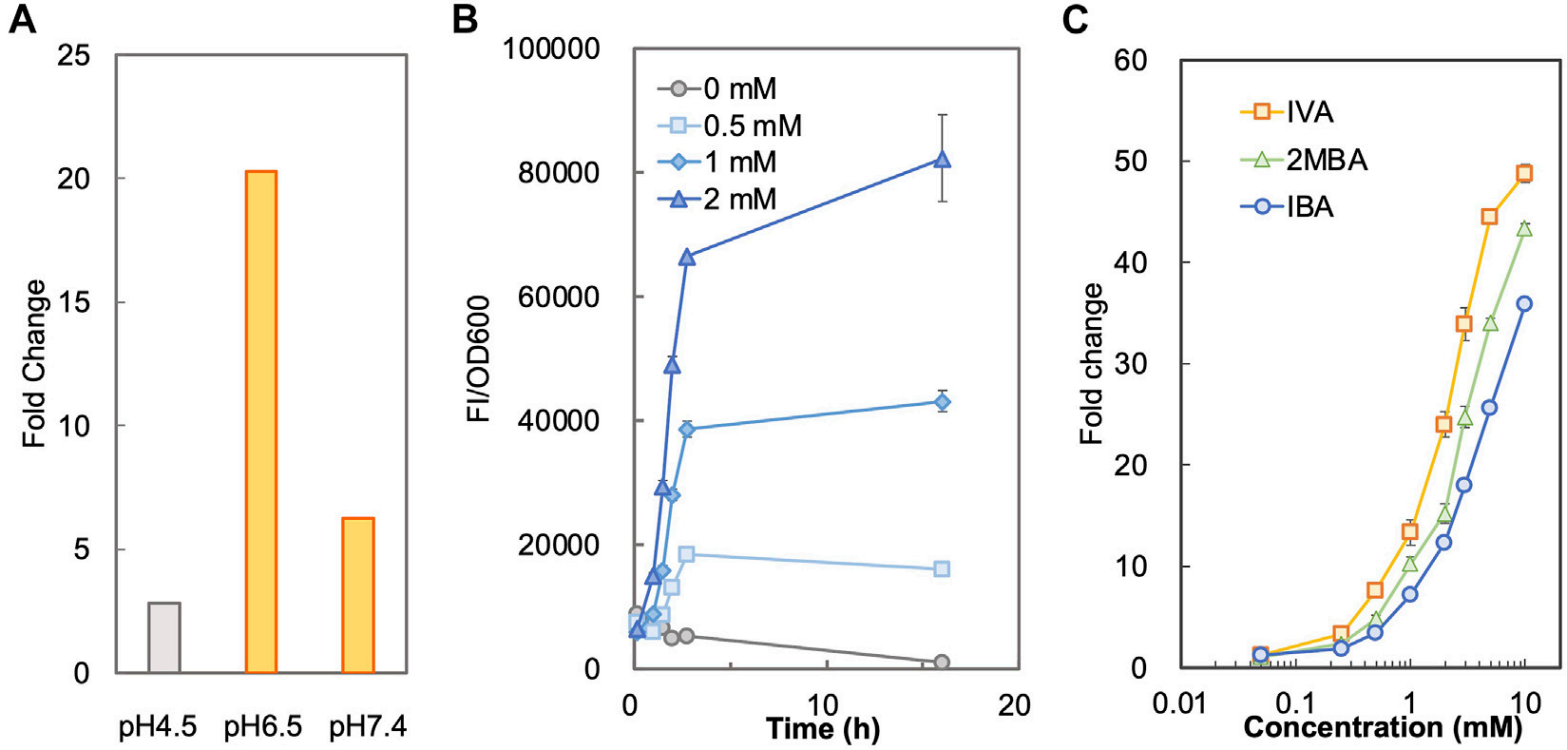
Characterization of biosensor BY4741-S_LCA_. **(A)** Responses of BY4741-S_LCA_ cells (fold changes) to IBA under different pH conditions. BY4741-S_LCA_ cells were grown in SCD-U medium under buffered (at pH6.5 and pH7.4) or unbuffered (at pH4.5) pH conditions. GFP fluorescence was measured after incubation with 1.0 mM IBA and normalized to cell density (OD600). The fluorescence of control without the IBA supply was normalized to its cell density and set to 1. **(B)** Time-course fluorescence intensity of BY4741-S_LCA_ cells in response to IBA supplied into the medium. BY4741-S_LCA_ cells were incubated in the buffered SCD medium (pH 6.5) with IBA supply (at 0.5, 1, and 2 mM). The fluorescence was measured and normalized to cell density (OD600). **(C)** Dose-dependent responses of BY4741-S_LCA_ cells to respective SBCFA, i.e. IVA, 2MBA and IBA after a 3-h incubation. FI, fluorescence intensity. Values are means of three independent experiments. An error bar represents a standard deviation of three independent experiments.

To determine a suitable condition for sensing SBCFAs, we measured and compared the fluorescence intensity of BY4741-S_LCA_ in SCD-U medium under buffered pH (at pH6.5 and pH7.4) and unbuffered (at pH4.5) pH conditions added with IBA and IVA, respectively. Supplementary Figure S2 shows that SLCA with a starting pH4.5 and unbuffered had a higher background than that when the pH was buffered to 6.5 and 7.4 without SBCFAs, suggesting that the low pH effect can be mitigated by maintaining the pH at about 7.0. The background fluorescence without pH buffering increased with incubation, whereas it remained low and constant under the buffered pH condition. It was found that under the unbuffered condition, the pH dropped from pH4.5 to pH3.5 after overnight incubation and under the buffered condition the pH remained above pH6.0. The higher fluorescence background under the unbuffered condition (pH 4.5) was likely due to the higher concentration of proton than that at pH 6.0. The undissociated weak organic acids can pass through the plasma membrane by passive diffusion under an acidic condition and free weak organic acids can be taken up by active transport at a physiological condition near pH7 (Casal et al., 2008). The uptaking efficiency of the former is generally more efficient than the latter (Borrull et al., 2015). We hypothesized that the whole-cell biosensor BY4741-SLCA would give a higher response at pH6.5 and pH7.4 than at pH4.5. This was proven by a 7.3-fold and 2.3-fold higher fold-change (FC) of fluorescence intensity towards IBA under the buffered pH at 6.5 (FC 20.3) and 7.4 (FC 6.3) than that when unbuffered (FC 2.8) at about 3 h (Figure 1A). Supplementary Figure S2B also shows that the time-course responses (FC) to IVA at pH6.5 (FC 37.0) and 7.4 (FC 14.9) are significantly higher than at pH4.5 (FC 3.9) at about 3 h. Given higher fluorescence from at pH6.5 than pH7.4, the medium buffered at pH6.5 was chosen for biosensing SBCFAs in this study.

Next, under the condition with the pH buffered to 6.5, we characterized the operational range of BY4741-S_LCA_ against individual SBCFAs (IVA, 2MBA, IBA) fed at various concentrations. We firstly determined the minimal incubation time required for the S_LCA_ to respond to the SBCFAs till the signal remained stable. Using IBA as an example, we found that there was a time-dependent exponential increase in the fluorescence signal against 0.5–2.0 mM of IBA till about 3 h. Afterwards, there was a slight decrease of signal from control (without IBA) (Figure 1B), which showed a 10-fold increase in cell density and a 2-fold increase in fluorescence (Supplementary Figure S3). This result suggests that a 3-h incubation is required prior to measuring the biosensor’s signal.

To determine the correlation of SBCFA doses to the signal, we exposed the BY4741-S_LCA_ to IVA, 2MBA and IBA fed at various concentrations (0.05–10 mM) and measured the fluorescence signal. In Figure 1C, with a 3-h incubation, BY4741-S_LCA_ demonstrated a dose-dependent increase of the response (FC) against the respective SBCFAs ranging from 0.05 mM (∼FC 2) to 10 mM (>FC 36). It is noted that, starting from 0.25 mM till 10 mM, the response from IVA was the highest (up to FC 49) amongst the three tested SBCFAs, suggesting that IVA is a preferred sensing target (EC_50_ 2.2) over 2MBA (up to FC 43, EC_50_ 3.6) and IBA (up to FC 36, EC_50_ 5.0). Our results show that our biosensor is suitable for screening SBCFA-overproducing strains that produce SBCFAs at 0.25–10 mM, including the engineered *S. cerevisiae* strain producing 4 mM SBCFAs (Yu et al., 2016).

Structurally, the branched chain of IBA (C4) and 2MBA (C5) is located at the α position, whereas IVA’s (C5) branched chain is at the β position further from the carboxylic group. Given the differences in the branched-chain positions, the binding affinity of War1p to these SBCFAs will differ, depending on the varying binding tendency towards the structurally dissimilar carbon chain. It is known that War1p prefers binding to lipophilic weak acids over hydrophilic weak acids (Kim et al., 2019). Therefore, the differences in the binding efficacy of War1p to the SBCFAs could cause different responses of the biosensor. In addition, intracellular concentration of these SBCFAs could vary due to different import/export efficiency, hence causing variable responses of the biosensor.

### Substrate Spectrum of the SBCFA Biosensor

Besides SBCFAs, we investigated the substrate spectrum of S_LCA_ against other representative industry-relevant short-chain weak organic acids, including short linear chain fatty acids and lipophilic carboxylic acid derivatives such as phenolic acids, methacrylic acid, hydroxy carboxylic acids, keto acid and branched-chain amino acid (Castano-Cerezo et al., 2019).

**FIGURE 2:**
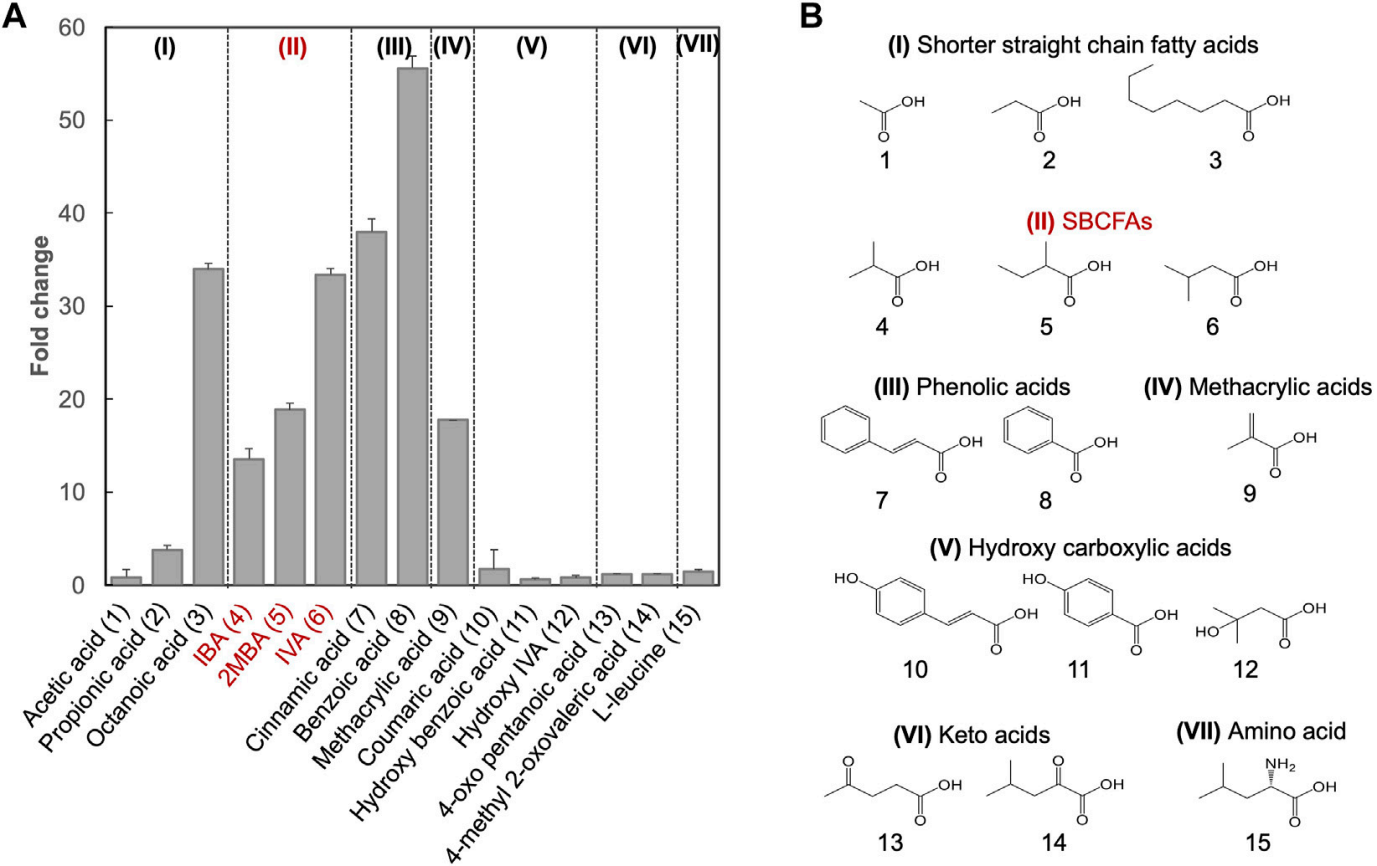
Substrate spectral analysis of biosensor BY4741-S_LCA_. **(A)** Responses of BY4741-S_LCA_ cells (fold change) to weak organic acids categorized based on the similarity of their chemical structures. GFP fluorescence was measured after overnight incubation at 30°C and normalized to cell density (OD600) and further to control without weak organic acid supply. Values are the means of three independent experiments, and an error bar represents a standard deviation of three independent experiments. **(B)** Molecular structures of the tested weak acids, comprising of shorter straight chain fatty acids (I), and lipophilic carboxylic acid derivatives including SBCFAs (II), phenolic acids (III), methacrylic acid (IV), hydroxy carboxylic acid (V), keto acids (VI) and amino acid Leucine (VII). Their numbers are indicated, and SBCFAs are highlighted in red.

To determine if shorter linear chain fatty acids and the lipophilic carboxylic acids with various chemical groups (vinyl, methyl, oxo, phenyl, hydroxyl and amino) were substrates of the biosensor BY4741-S_LCA_, we investigated its response to acetic (C2), propionic (C3) and octanoic (C8) acids and several lipophilic carboxylic acid derivatives (Figure 2). We found that S_LCA_ was responsive to propionic acid (FC 3.8) and octanoic acid (FC 34.0), but not acetic acid. This result suggests that the pPDR12-based genetic biosensor respond to linear chain fatty acids with 3 carbons (C3) and longer. In addition, the biosensor S_LCA_ showed a strong response (FC 40.0), consistent with a previous study (Baumann et al., 2018).

For methacrylic acid, a lipophilic carboxylic acid derivative with a vinyl group, a significant response was observed from the biosensor (FC 17.7). In line with previous studies (Hatzixanthis et al., 2003; Kren et al., 2003), the biosensor showed an FC 55.6 in response to benzoic acid (with a phenyl group) and an FC 38.0 in response to cinnamic acid (with a phenyl and a vinyl group). To our knowledge, this is the first evidence on sensing cinnamic acid using the pPDR12-based system in *S. cerevisiae*. We did not observe any responses (FC < 2.0) to the SBCFA’s precursors including L-leucine (with an amino group) and 4-methyl 2-oxovalerate (with a methyl and an oxo group), as well as hydroxylated derivatives including hydroxy isovaleric acid, hydroxy benzoic acid and coumaric acid. Supplementary Table S2 shows that some carboxylic acids (e.g., hydroxy isovaleric acid and 4-oxo pentanoic acid) have a relatively low hydrophobic constant (logP), i.e., a relatively low solubility in the medium. Interestingly, coumaric acid and hydroxy benzoic acid have high logP values. The lack of response to these compounds might be due to their low intracellular concentration caused either by low uptake, high efflux, or cellular metabolism (Barnhart-Dailey et al., 2019).

### Optimization of the SBCFA Biosensor by *PDR12* Deletion and Overexpression

Given the role of transporters in regulating the intracellular level of SBCFAs (Piper et al., 1998; Nygard et al., 2014), we examined the effect of deleting and overexpressing an efflux pump gene *PDR12* on the S_LCA_’s behaviors (sensitivity and operational range). We hypothesized that the deletion of *PDR12* would result in a higher intracellular level of SBCFAs than that in the wild-type cells and *vice versa* for *PDR12* overexpression.

**FIGURE 3:**
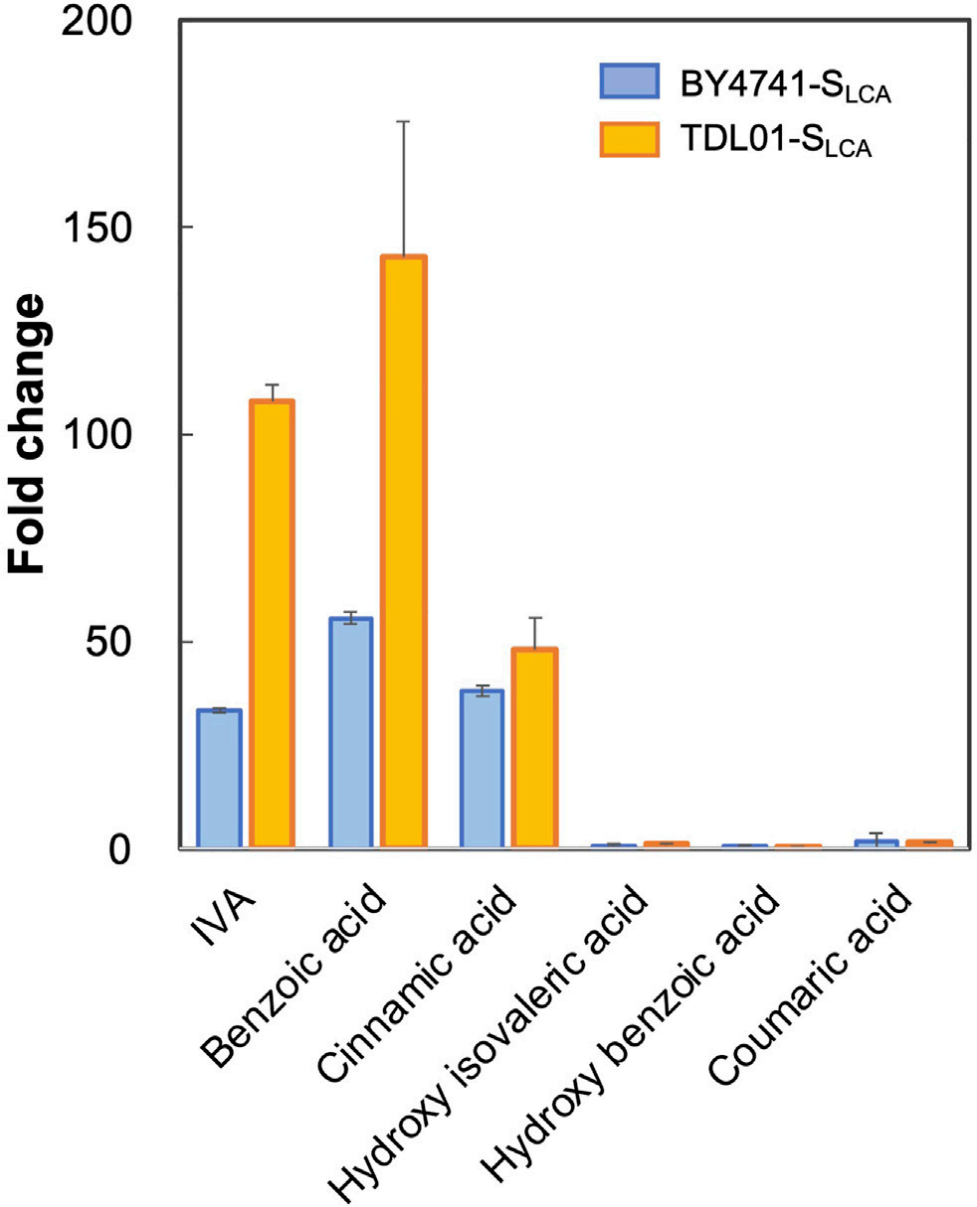
Responses of biosensor TDL01-S_LCA_ with *PDR12* gene deletion to representative weak organic acid including IVA, benzoic acid, cinnamic acid, hydroxy isovaleric acid, hydroxy benzoic acid and coumaric acid. BY4741-S_LCA_ and TDL01-S_LCA_ cells were grown until the exponential phase. After a 16-h incubation in a buffered SCD-U medium (at pH6.5) with the supply of weak organic acids (2 mM each), GFP fluorescence was measured, and fold changes were calculated. Values are means of three independent experiments, and an error bar represents standard deviation of three independent experiments.

**FIGURE 4:**
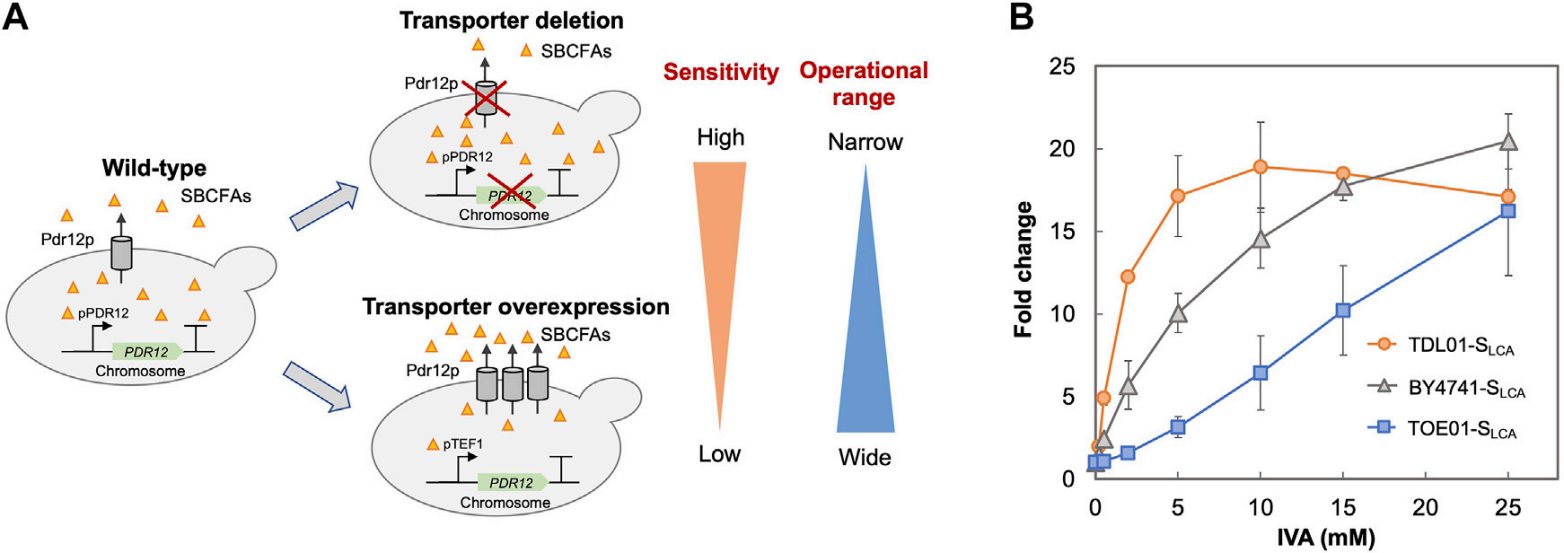
Optimization of biosensor’s sensitivity and operational range. **(A)** Schematic presentation of strategies employed to optimize the biosensor. The ABC transporter Pdr12p is known to regulate the intracellular level of weak organic acids by export. To improve the biosensor’s sensitivity, an ABC transporter gene *PDR12* was deleted to increase the intracellular SBCFA level (TDL-S_LCA_), which will increase the fluorescence. To extend the biosensor’s operational range, *PDR12* was over-expressed under the control of a strong constitutive promoter pTEF1 to reduce the intracellular SBCFA level (TOE-S_LCA_) through SBCFA export, which will enhance the SBCFA saturation level. **(B)** Dose-dependent responses of biosensors against IVA fed at 0.5, 2, 5, 10, and 25 mM. Their responses (fold change) were compared to control BY4741-S_LCA_. Fluorescence was measured after a 5-h incubation with IVA. Values are the means of three independent experiments, and an error bar represents a standard deviation of three independent experiments.

Based on the above hypothesis, we sought to enhance the biosensor’s sensitivity through *PDR12* deletion and to shift the operational range through *PDR12* overexpression (Figure 4A). Firstly, we deleted *PDR12* gene (Supplementary Figure S1) and examined its effect on cell growth in the presence of 15 mM IVA. We found that the *PDR12*-deficient strain (TDL01) had a slight growth retardation after 12 h in the absence of IVA. In the presence of IVA, TDL01 showed a significant growth retardation till the log-phase when it had a cell density comparable to its parental strain BY4741 (Supplementary Figure S4). The significant growth retardation suggests a growth inhibition caused by the higher intracellular IVA accumulated in TDL01. Secondly, we introduced the biosensor plasmid pPDR12-GFP into TDL01 and measured the FC of the resulting strain TDL-S_LCA_. TDL-S_LCA_ was found to have a response of FC 107.9 to 2 mM IVA, which is over 3-fold higher than BY4741 (FC 33.4). The increase in response to IVA in TDL-S_LCA_ confirms the significant improvement in sensitivity. In addition, there was an increase in response by about three times to 2 mM of benzoic acid and a slight increase against cinnamic acid. We observed no change in the response toward other carboxylic acids (i.e., hydroxyl isovaleric acid, hydroxyl benzoic acid and coumaric acid) (Figure 3). These results show that the *PDR12* deletion improves the sensitivity of the pPDR12-GFP biosensor to its target sensing substrates. Thirdly, to evaluate the operational range of the biosensor, we investigated its response to IVA at various doses in the *PDR12*-deficient (TDL01) and -overexpressing (TOE01) strains (Figure 4B and Supplementary Figure S1). Given a higher expression level of *PDR12* upon the replacement of pPDR12 with the TEF1 promoter (pTEF1) in *S. cerevisiae* (Yu et al., 2016), in the absence of exogenous IVA supply, the biosensor TOE01-S_LCA_ showed the lowest background fluorescence in SCD medium (Supplementary Figure S5). TOE01-S_LCA_ showed a shift of the operational range towards higher concentrations to exogenous IVA (2–25 mM) compared to BY4741-S_LCA_ (Control). Given that the response was not saturated at 25 mM IVA, there would be a continuing increase of the response to IVA at >25 mM. Thus, we speculated an operational range that is wider than 2–25 mM. TDL01-S_LCA_ showed a higher sensitivity (up to FC18.9) toward exogenous IVA (0.2–10 mM). EC_50_ of TDL01-S_LCA_ (1.3 mM) was 7.7-fold lower than that of BY4741-S_LCA_ (10 mM).

The higher sensitivity (TDL01-S_LCA_) and wider operational range (TOE01-S_LCA_) could be due to changes in export efficiency caused by *PDR12* overexpression and deletion. Our results suggest that maintenance of the appropriate intracellular level of target biochemical can be used to enhance the operational range or widen the sensitivity of a whole-cell biosensor. Whole-cell biosensors with various operational ranges or sensitivities are desired for pathway engineering and strain optimization. For instance, at the early stage of pathway engineering when the production level of target biochemical is low, a biosensor with a higher sensitivity like TDL01-S_LCA_ is favored for screening strain screening. On the other hand, at a later stage, a biosensor with a wider operational range like TOE01-S_LCA_ is suitable for optimizing the production to achieve a higher production level.

### Sensing of SBCFAs in the SBCFAs-Overproducing Strain

After we studied S_LCA_’s responses to exogenously supplied SBCFAs, we used it to sense SBCFAs in an SBCFA-overproducing strain. We previously engineered *S. cerevisiae* BY4741 (4G-ΔADH6) to overproduce SBCFAs at up to 3.7 mM SBCFAs (Yu et al., 2016). Given that the intracellular level of SBCFAs lies within the identified operational range of TDL01-S_LCA_ (0.2–10 mM), we employed the strategy of *PDR12* deletion for the biosensing. To this end, we deleted *PDR12* in 4G-ΔADH6, resulting in TDL02. Next, we introduced the pPDR12-GFP plasmid into 4G-ΔADH6, TDL02 and analyzed the fluorescence signals of the resulting strains 4G-ΔADH6-S_LCA_ and TDL02-S_LCA_.

**FIGURE 5:**
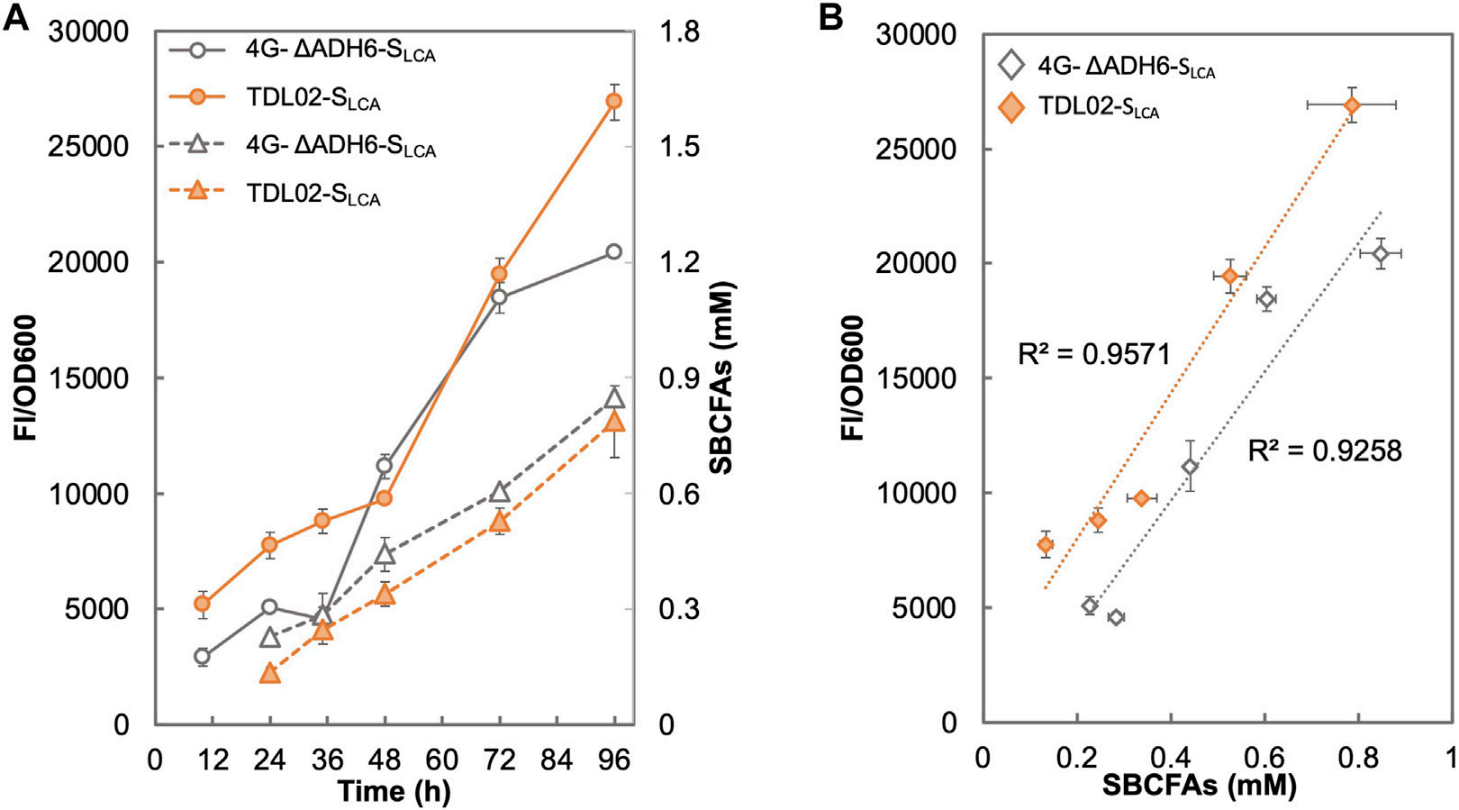
Responses of SBCFA biosensor in a *S. cerevisiae* strain overproducing SBCFAs. *PDR12* was deleted in a previously engineered SBCFA-overproducing strain 4G-ΔADH6, resulting in strain TDL02. The biosensor S_LCA_ was introduced into TDL02 and 4G-ΔADH6 respectively. **(A)** The fluorescence (solid lines) and the over-produced SBCFAs (dotted lines) were measured after fermentation in buffered SCD-U (at pH6.5) medium for up to 96 h. **(B)** Correlation between GFP fluorescence with SBCFAs produced in 4G-ΔADH6-S_LCA_ and TDL02-S_LCA_. Trendlines with respective *R* ^2^ value are shown in orange (4G-ΔADH6-S_LCA_) and grey (TDL02-S_LCA_). Values are the means of three independent experiments, and an error bar represents a standard deviation of three independent experiments.

Figure 5A shows that the fluorescence signal of 4G-ΔADH6-S_LCA_ intensified with the increase in SBCFA production. The fluorescence signal of TDL02-S_LCA_ also showed an increase along with the SBCFA production, but up to 76.7% higher than 4G-ΔADH6-S_LCA_. The fluorescence signal of both 4G-ΔADH6-S_LCA_ and TDL02-S_LCA_ showed a linear correlation to the produced SBCFAs (*R* ^2^ > 0.93) (Figure 5B). Given that the SBCFA production level is comparable in 4G-ΔADH6 and TDL02, the fluorescence signal suggests the biosensor S_LCA_ can monitor the SBCFA production real-time and the *PDR12* deletion enhances the sensitivity of the biosensor in the SBCFA-overproducing strain. The sensitivity enhancement is consistent with BY4741 and TDL01 with SBCFA supplementation. At 48 h, the signal of TDL02-S_LCA_ is slightly lower than 4G-ΔADH6-S_LCA_, likely due to a slightly lower SBCFA production in TDL02-S_LCA_ (0.34 mM) than 4G-ΔADH6-S_LCA_ (0.44 mM) (Supplementary Figure S6).

In terms of the biosensing substrates, TDL02-S_LCA_ and 4G-ΔADH6-S_LCA_ sensed the mixture of SBCFAs, where the fluorescence signal attributes to the responses from such a mixture of SBCFAs. However, the contribution of each SBCFA in the mixture to the fluorescence is still unclear. In addition, we observed a 3-fold lower SBCFA titer in both 4G-ΔADH6 and TDL02 and a lower proportion of IVA/2MBA than those reported in our previous study (Yu et al., 2016). Such changes in the production titer and SBCFA profiles could be due to the use of a defined medium SCD that allows the SBCFA production from glucose, whereas in our previous study, the SBCFAs were likely produced from glucose and branched-chain amino acids present in YPD medium. Nevertheless, the SBCFA levels obtained using SCD were shown to lie within the operational range of our biosensor TDL01-S_LCA_ which allows real-time monitoring of the SBCFA production in the engineered strains.

## Conclusion

In this study, we engineered a genetic biosensor based on War1p and pPDR12. The biosensor detected SBCFAs (e.g., IVA, IBA and 2MBA) that were exogenously fed and intracellularly produced by engineered *S. cerevisiae* strains. The biosensor’s properties including sensitivity and operational range were optimized by varying the expression level of *PDR12* encoding an ABC transporter responsible for SBCFA export. Besides SBCFAs, the biosensor can also sense short linear chain fatty acids and lipophilic carboxylic acid derivatives. Our findings suggest that the engineered biosensor is potentially a useful tool for monitoring the production of biochemicals, such as IVA, IBA, 2MBA, benzoic acid, cinnamic acid, propionic acid and octanoic acid, and for screening and discovering high producer strains.

Given the low sensitivity observed when the SBCFAs were at low concentrations (<0.25 mM), future efforts could be focused on improving the sensitivity. One strategy could be to introduce a positive-feedback loop on War1p to enhance the sensitivity. Williams et al. reported that the promoter replacement of the *WAR1* promoter with pPDR12 in the genome, where War1p regulates its own expression in response to propionic acid (Williams et al., 2017), resulting in a dramatic increase in the sensitivity (from FC 3.6 to FC 10). In our current study, we confirmed an operational range of up to ∼0.8 mM of the intracellularly produced SBCFA mixture. To effectively detect SBCFAs produced by a higher producer, the operational range could be further evaluated and improved through optimization of the binding affinity between SBCFAs and the receptor domain of War1p, DNA binding domain of War1p or WARE sequence of pPDR12 (Gregori et al., 2008).

## Data Availability

The original contributions presented in the study are included in the article/Supplementary Material, further inquiries can be directed to the corresponding author.
