# Supplementary material for: Transporter-Driven Engineering of a Genetic Biosensor for the Detection and Production of Short-Branched Chain Fatty Acids in Saccharomyces cerevisiae
Source: Front Bioeng Biotechnol. 2022 Mar 18;10:838732. doi: 10.3389/fbioe.2022.838732 (PMC8975619; doi:10.3389/fbioe.2022.838732)
Supplement: Supplementary file 1 [file DataSheet1.pdf]

Transporter-driven engineering of a genetic biosensor for the detection and production of short-branched chain fatty acids in *Saccharomyces cerevisiae*

Ryoma Miyake[1,2,3,4], Hua Ling[1,2,3], Jee Loon Foo[1,2,3], Nobutake Fugono[4], Matthew Wook Chang[1,2,3*]

[1] NUS Synthetic Biology for Clinical and Technological Innovation (SynCTI), National University of Singapore, Singapore 117456, Singapore

[2] Synthetic Biology Translational Research Programme, Yong Loo Lin School of Medicine, National University of Singapore, Singapore 117456, Singapore

[3] Department of Biochemistry, Yong Loo Lin School of Medicine, National University of Singapore, Singapore 117597, Singapore

[4] Science & Innovation Center, Mitsubishi Chemical Corporation, Yokohama-shi, Kanagawa 227-8502, Japan

*** Correspondence:**

Matthew Wook Chang, Tel: +65 6601 3687, E-mail: bchcmw@nus.edu.sg

Supplementary tables

Supplementary Table 1. Sequences of primers used in this study

Name	Sequence	Purposes
PDR12p-f	CGAAGAATTGTTAATTAAGAGCTGATATC TTTGTTTTGCATTTTAC	pPDR12 amplification
PDR12p-r	GAATAATTCTTCACCTTTAGACATTTTTTTA TTAATAAGAACAATAAC	
yEGFP-f	ATGTCTAAAGGTGAAGAATTATTC	yEGFP gene amplification
yEGFP-r	GCCGCGGTACCAAGCTTACTCGAGTTATTT GTACAATTCATCCATACCA	
PDR12-Del-f	GATTATGCTAATTCGGTACAATCCTACGCTG CCTCCGAAGGCCTGAAGCTTCGTACGCTG	PDR12 gene deletion
PDR12-Del-r	ATCCCAAGAGTAGATGGAGGCATTCATTGCC TGAGCTTCAACAGGCCACTAGTGGATCTG	
PDR12-CheGD-F	ATGTCTTCGACTGACGAACA	
PDR12-CheGD-R	CGTTCTAATGGCTTGGGCAA	
PDR12-pTEF1-F	CGTTAAGAGCCGATTCGCCGGAATTTCTCG ATTTTTTTCCGTGCAGGTCGACAACCCTTA	pPDR12 replacement
PDR12-pTEF1-R	CGACGAAATGTCTTTCTCAATATGTTCGTCA GTCGAAGACATTTTGTAATTAAAACTTAG	
TEF1p-UP-F	AGGTGATATCAGATCCACTAGT	
PDR12-CheTEFp-r	GCATAATCATCGTCATGGTTCG	

Supplementary Table 2. Chemical and physical properties of carboxylic acids tested in this study

**Carboxylic acids**	**pKa**	**LogP**	**PubChem CID****
Cinnamic acid	4.55*	2.13	444539
Benzoic acid	4.19	1.87	243
Octanoic acid	4.89	3.05	379
Isovaleric acid	4.77	1.16	10430
2-methylbutyric acid	4.80*	1.18	8314
Isobutyric acid	4.84	0.94	6590
Methacrylic acid	4.65	0.93	4093
Propionic acid	4.88	0.33	1032
Acetic acid	4.76	-0.17	176
Coumaric acid	4.58*	1.79	637542
4-hydroxy benzoic acid	4.54	1.58	135
4-Hydroxyisovaleric acid	4.38*	-0.35*	69362
4-Oxopentanoic acid	4.64	-0.49	11579
L-leucine	2.36	-1.52	6106
4-methyl 2-oxovaleric acid	2.65	0.9	70

* Calculated using Advanced Chemistry Development (ACD/Labs) Software V11.02.

**Data sources are available at https://pubchem.ncbi.nlm.nih.gov/. pKa, acid dissociation constant, LogP, partition coefficient.

Supplementary Figures

Supplementary Figure 1

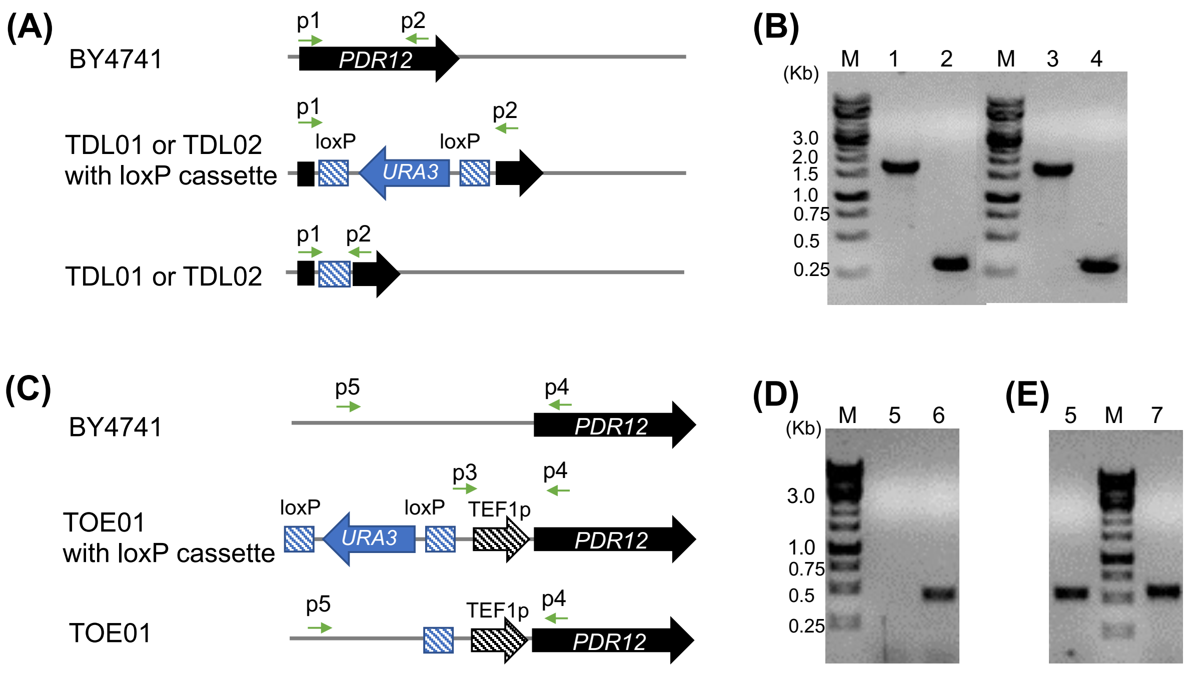

Supplementary Figure 1. Genetic modifications of *S. cerevisiae* strains. *PDR12* gene was deleted through homologous recombination and Cre-LoxP-based recovery to create TDL01 and TDL02 (A) which were confirmed by PCR amplification of target genomic DNA fragments using primer p1 and p2 (B). *PDR12* gene was over-expressed by replacing its native promoter with TEF1 promoter through homologous recombination and Cre-LoxP-based recovery to create TOE01 (C) confirmed by PCR amplification of target genomic DNA fragments using primer p3 and p4 (D) and using p4 and p5 (E). M, a DNA ladder with band sizes indicated, lane 1-4, TDL-01 before and after removing a loxP cassette, TDL-01 before and after *URA3* marker rescue, TDL02; lane 5-6, BY4741, TOE01 after *URA3* maker rescue.

Supplementary Figure 2

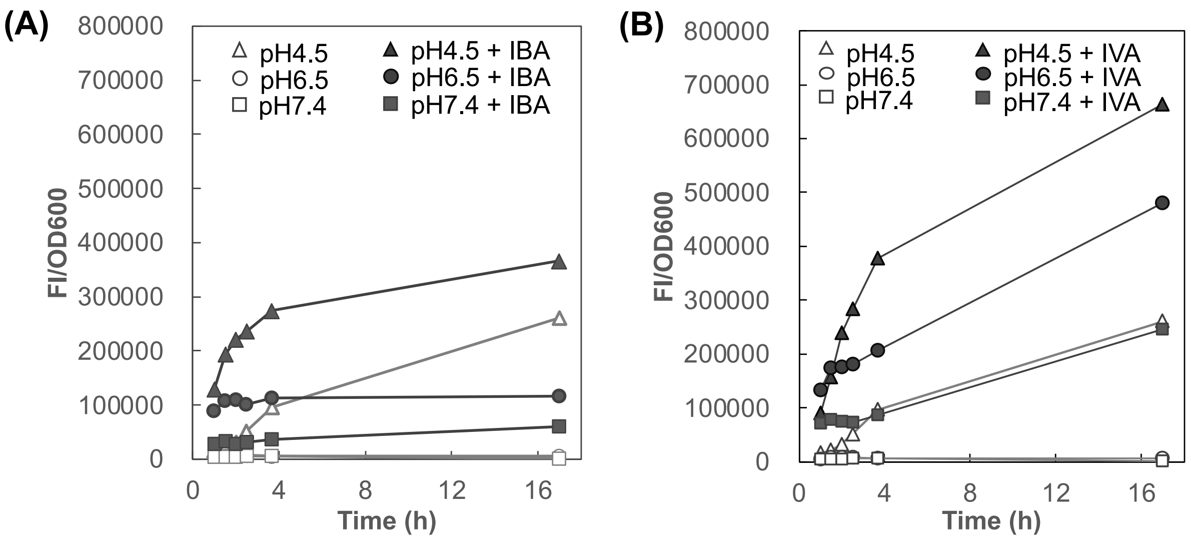

Supplementary Figure 2. Time-course fluorescence intensity of BY4741-S_{LCA} in response to IBA (A) and IVA (B), respectively in unbuffered (at pH4.5) and buffered (at pH6.5 and pH7.4) SCD medium. BY4741-S_{LCA} was cultivated in SCD medium fed with 1 mM IBA or IVA at 30 °C. The fluorescence was measured accordingly.

Supplementary Figure 3

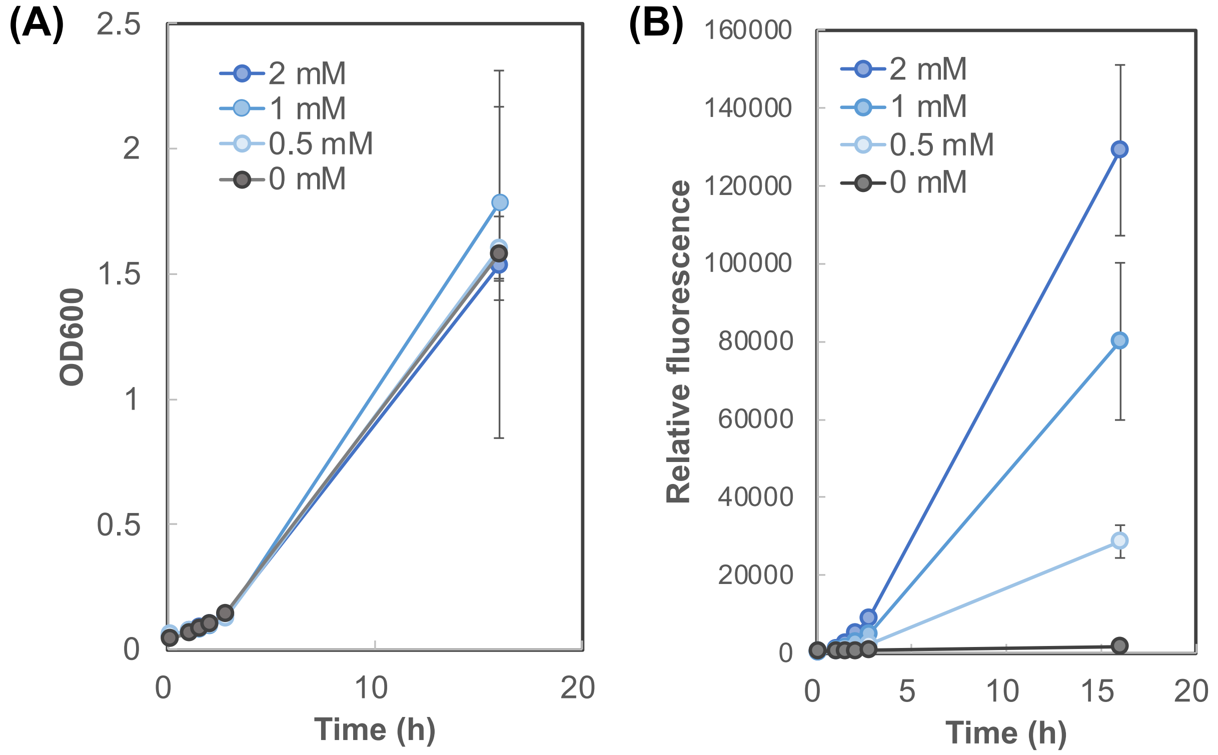

Supplementary Figure 3. Time-course cell density (A) and relative fluorescence (B) of BY4741-S_{LCA} cells in response to IBA supplied into the medium. BY4741-S_{LCA} cells were incubated in the buffered SCD medium (pH6.5) with IBA supply (at 0.5, 1, and 2 mM).

Supplementary Figure 4

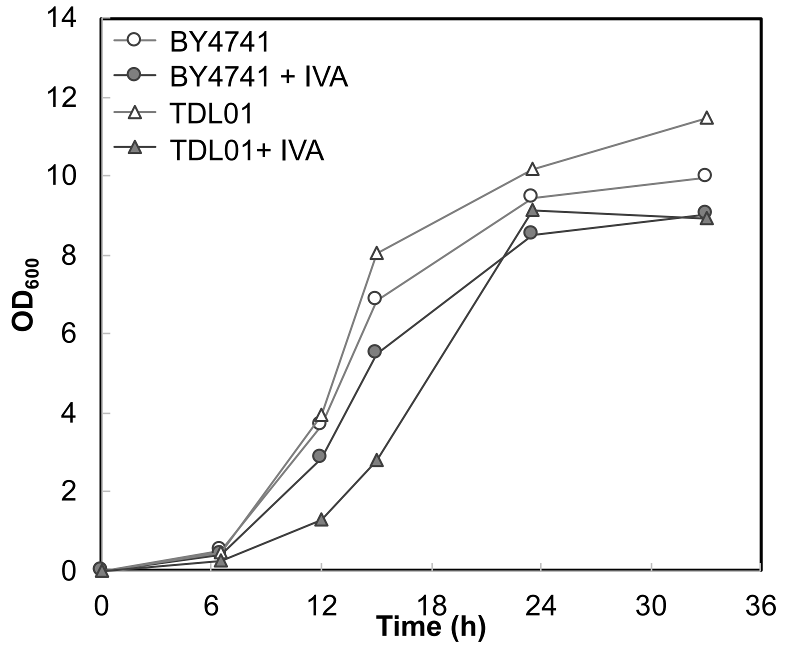

Supplementary Figure 4. Growth profiles of TDL01 and BY4741 in the buffered SCD medium (at pH6.5) with and without the supply of 15 mM IVA.

Supplementary Figure 5

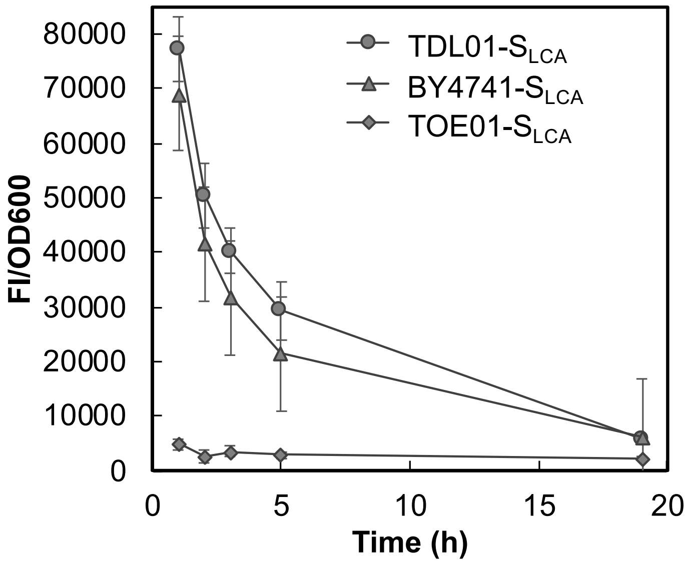

Supplementary Figure 5. Time-course background fluorescence of biosensor strains. The strains were cultivated in SCD medium without SBCFA supply. The fluorescence was measured and normalized to their cell density (OD_{600}). Values are the means of three independent experiments. An error bar represents a standard deviation of three independent experiments.

Supplementary Figure 6

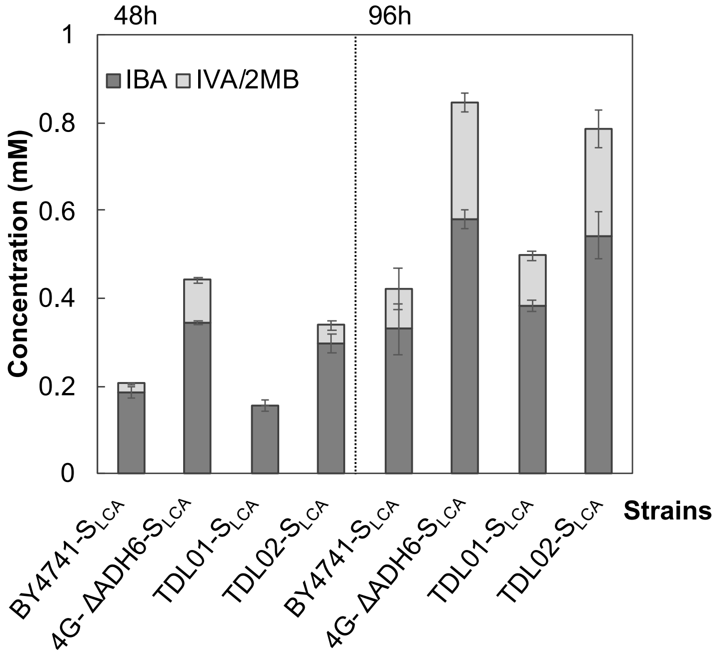

Supplementary Figure 6. Composition of SBCFAs in the engineered *S. cerevisiae* producing SBCFA. Values are the means of three independent experiments. An error bar represents a standard deviation of three independent experiments.
